# Case-crossover study to examine the change in postpartum risk of pulmonary embolism over time

**DOI:** 10.1186/s12884-017-1283-y

**Published:** 2017-04-14

**Authors:** Grégoire Ficheur, Alexandre Caron, Jean-Baptiste Beuscart, Laurie Ferret, Yu-Jin Jung, Charles Garabedian, Régis Beuscart, Emmanuel Chazard

**Affiliations:** 1 Department of public health, Lille University Hospital, EA 2694 - Public health: Epidemiology and quality of care, 2 Avenue Oscar Lambret, F-59000 Lille, France; 2 Department of pharmacology and clinical pharmacy, Lille University Hospital, 2 Avenue Oscar Lambret, F-59000 Lille, France; 3 Department of Obstetrics, Lille University Hospital, Jeanne de Flandre Hospital, 2 Avenue Oscar Lambret, F-59000 Lille, France

## Abstract

**Background:**

Although the current guidelines recommend anticoagulation up until 6 weeks after delivery in women at high risk of venous thromboembolism (VTE), the risk of VTE may extend beyond 6 weeks. Our objective was to estimate the risk of a pulmonary embolism in successive 2-week intervals during the postpartum period.

**Methods:**

In a population-based, case-crossover study, we analyzed the French national inpatient database from 2007 to 2013 (*n* = 5,517,680 singleton deliveries). Using ICD-10 codes, we identified women who were diagnosed with a postpartum pulmonary embolism between July 1^st^, 2008, and December 31^st^, 2013. Deliveries were identified during a case “period” immediately before the pulmonary embolism, and five different control periods one year before the pulmonary embolism. Using conditional logistic regression, Odds ratios (ORs) and 95% confidential intervals (CIs) were estimated for ten successive 2-week intervals that preceded the diagnosis of pulmonary embolism.

**Results:**

We identified 167,103 cases with a pulmonary embolism during the inclusion period. After delivery, the risk of pulmonary embolism declined progressively over time, with an OR [95%CI] of 17.2 [14.0–21.3] in postpartum weeks 1 to 2 and 1.9 [1.4–2.7] in postpartum weeks 11 to 12. The OR [95%CI] in postpartum weeks 13 to 14 was 1.4 [0.9–2.0], and the OR did not fall significantly after postpartum week 14.

**Conclusions:**

Our findings indicate that women are at risk of a pulmonary embolism up to 12 weeks after delivery. The shape of the risk curve suggests that the risk decreases exponentially over time. Future research is needed to establish whether the duration of postpartum anticoagulation should be extended beyond 6 weeks.

**Electronic supplementary material:**

The online version of this article (doi:10.1186/s12884-017-1283-y) contains supplementary material, which is available to authorized users.

## Background

The postpartum period is associated with an elevated risk of a venous thromboembolic event (VTE). The American College of Chest Physicians recommends that patients at high risk of thromboembolism should receive prophylactic anticoagulation therapy for 6 weeks following delivery [1]. In France, recommendations for prophylactic anticoagulation are similar [2]. However, based on the results of four studies [3–6], it is not clear whether the risk of VTE extends beyond 6 weeks postpartum. Studies by Ros et al. [3] and Heit et al. [4] (estimated by Jackson et al. [7] from reported data) did not find an elevated risk of VTE after 6 weeks, whereas studies by Pomp et al. [5] and Kamel et al. [6] evidenced an elevated risk for at least 12 weeks after delivery. Indeed, the most detailed of these studies (with 3-week time intervals) concluded that an elevated risk could extend up to 15 weeks postpartum [5].

In light of these findings, we decided to explore the relative risk of a postpartum VTE with a greater degree of precision. It is noteworthy that studies reporting incidence rates (without any assessment of the relative risk) give estimations for week-long intervals [4, 5, 8, 9]. Hence, a large population-based study of how the risk of a postpartum VTE decreases over time after delivery was warranted.

### Objective

The objective of the present study was to assess the risk of a postpartum VTE in 2-week time epochs extending from the date of delivery.

## Methods

### Data sources

Collection of the study data was approved by the French National Data Protection Commission (CNIL; authorization number: 1754053). The “acute care” section of the French national inpatient database contains information on 171,556,421 inpatient stays and 5,517,680 singleton deliveries linked to 4,252,507 mothers between January 1^st^, 2007 to December 31^st^, 2013. Summary data for each inpatient stay in an “acute care” department of a public -or private- sector hospital are collected by the French National Health Insurance Agency (*Assurance Maladie* [10]). The corresponding database contains the ICD-10 diagnostic codes [11], the medical procedures performed (coded according to the French national “CCAM *Classification Commune des Actes Médicaux*” classification), and the patient’s age, gender, and unique identifier. The time interval between two different hospital admissions are expressed as the number of days.

A total of 153 data completeness and coherence checks [12] are performed routinely when the information on inpatient admissions is sent to the French National Health Insurance Agency. These include checks on the chronology of the inpatient admissions, data integrity/accuracy (i.e. missing, incorrect or imprecise values) of patient’s and admissions’ data (gender, age, date and mode of entry, and date and mode of discharge), the format of the procedure codes and the diagnostic codes, and the concordance between procedure codes, diagnostic codes, the length of stay, and patients’ age and gender. More specifically, consistency controls between the following duplets are conducted: “procedure or diagnosis” AND “the antepartum/postpartum period”; “procedure or diagnosis” AND “gestational age”; “diagnosis on delivery” AND “procedure on delivery”.

From March 1^st^, 2009, the code for the “primary diagnosis” corresponds to the primary medical indication for hospital admission, and is recorded at the time of the patient’s discharge. Emergency room visits that did not result in an inpatient hospital admission are not included in the database.

### Study design

**Fig. 1:**
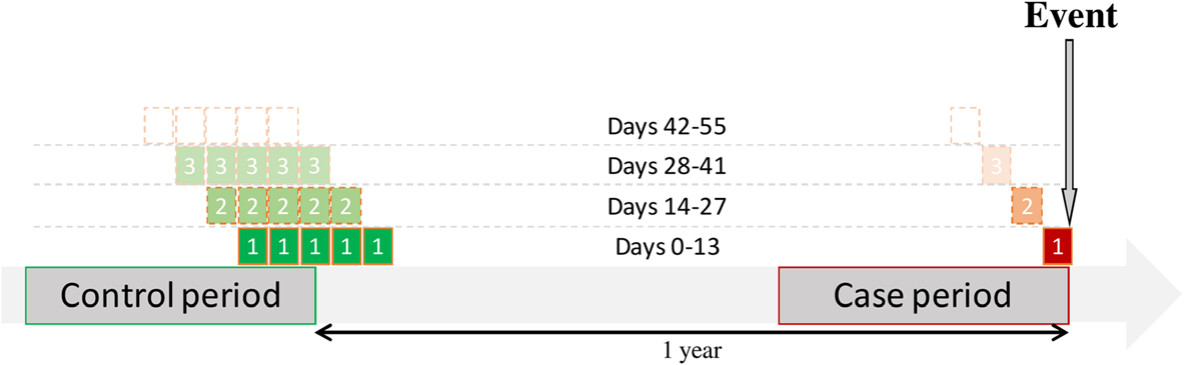
Illustration of a case-crossover design with five control periods

We carried out a population-based, case-crossover study. The Observational Medical Outcomes Partnership (OMOP, which promotes an empirical, strict, systematic evaluation of study designs) has shown that a crossover design is appropriate for the type of objective set out in the present study [13]. Each patient served as her own control, which enabled us to control for personal, time-constant confounding factors. The patient is analyzed immediately before a thromboembolic event and then used as her own control 12 months before (i.e. far from the thromboembolic event). Thus, for the whole set of patients (Fig. 1), we compared the likelihood of having had an inpatient stay for delivery in the 20 weeks preceding the VTE with the likelihood of having had an inpatient stay for delivery during five different time periods around a year before the occurrence of the VTE. Our use of five different control periods long before the VTE not only provided reasonable statistical power but also increased the number of deliveries during the control period (and thus decreased the variability of the mean during that period). Using a control period in the past is often necessary in a case-crossover design because it minimizes survival bias; given that a woman who has suffered from a thromboembolic event is less likely to get pregnant afterwards. Therefore, the control period must precede the case period.

### Patients

#### Inclusion criteria (definition of a case)

We identified inpatient admissions for a VTE between July 1^st^, 2008, to December 31^st^, 2013. If a patient suffered more than one VTE during that period, only the first-occurring event was analyzed.

Several algorithms for tracking VTEs in claims data have been developed and evaluated [14]. Many of these algorithms refer to ICD-9 codes. In a study by Casez et al., ICD-10 [15] discharge diagnosis codes were found to be sensitive tools for identifying pulmonary embolism (Sensitivity = 88.9% [85.6%–92.2%]) but not deep vein thrombosis (Sensitivity = 58% [51.9%–64.1%]). Furthermore, deep vein thrombosis does not necessarily require inpatient hospital admission. Accordingly, we chose pulmonary embolism as a marker of the risk of a VTE in the present crossover study.

The ICD-10 codes used to identify pulmonary embolism are detailed in the Additional file 1, as are the exclusion criteria.

### Measurements

#### Definition of exposure

Deliveries (i.e. the exposure of interest) for patients aged between 15 and 45 years (at the time of delivery) were identified during the case period and the control periods.

Inpatient delivery hospitalizations were classified by the presence of the diagnosis code Z37.0 (“Single live birth”) and the absence of the diagnosis codes O35.x (“Maternal care for known or suspected fetal abnormality and damage”) and O28.x (“Abnormal findings on antenatal screening of mother”). According to French guidelines [16], the diagnosis code Z37.0 must be entered for all single, live births. A prior comparison with national birth records has confirmed that the French national inpatient database’s use of this diagnosis code is reliable [17].

#### Retrospective calculation of the time interval between pulmonary embolism and delivery

The CCAM classification enabled us to identify all delivery procedures and the exact date of each delivery. We separated these events into two classes, according to whether the pulmonary embolism occurred during a later postpartum hospitalization or during the delivery hospitalization. In the former instance, we used the later admission date to calculate the time interval between delivery and the pulmonary embolism. In the latter instance (which was less frequent), calculating the time interval was more complicated. We had to (i) confirm that the VTE had occurred after the delivery and (ii) estimate the time interval between the delivery and the event. Firstly, the “primary diagnosis” (i.e. the reason for inpatient hospital admission) had to be compatible with a delivery, in order to confirm that the pulmonary embolism had occurred after delivery. Secondly, the dates of procedures (CCAM codes presented in the Additional file 1) required to diagnose pulmonary embolism enabled us to calculate the time interval.

### Statistical analysis

Firstly, we performed a descriptive analysis of all singleton, live births between 2007 and 2013. Categorical and binary variables were expressed as frequencies. Continuous data were expressed as the mean ± standard deviation (SD). Based on previously published data, we studied the following risk factors for postpartum VTE [18]: pre-term delivery (delivery ≤37 weeks’ gestation), maternal age > 35 years, preeclampsia or eclampsia, morbid obesity (BMI ≥40 kg/m^2^), cesarean section, postpartum hemorrhage, postpartum infection, and birth weight <2.5 kg.

Secondly, we calculated the number of pulmonary embolisms for each 14-day interval for the case period and for the control periods. The pulmonary embolism rate for 100,000 deliveries was also computed by using the number of deliveries between July 1^st^, 2008, and December 31^st^, 2013, as the denominator. Next, we compared the likelihood of a delivery occurring from 0 to 13 days before a pulmonary embolism with the likelihood of a delivery occurring during five, 2-week-long control periods: from day 330 to day 343 before the thromboembolic event, from day 344 to day 357, from day 358 to 371, from day 372 to 385, and from day 386 to day 399. We performed the same analysis ten times (using the case-crossover approach) for each 2-week interval before the pulmonary embolism. We used conditional logistic regression to calculate the odds ratio (OR) and the 95% confidence interval (CI) for each 2-week interval. An additional analysis was performed by stratifying by the mode of delivery; this enabled us to determine whether the risk was higher after cesarean section than after vaginal delivery. These results are presented in the Additional file 1.

A post-hoc conservative analysis was also performed by computing the risk after the exclusion of (i) cases with a length of stay (for delivery) greater or equal to 10 days (*n* = 126) and (ii) cases with an intercurrent admission between the time of the inpatient stay for delivery and the time of the inpatient stay for pulmonary embolism (*n* = 112).

Lastly, we implemented a negative control by assessing exposure that was not expected to lead to an elevated risk of a VTE. To this end, we analyzed the CCAM code AHPA009 (“Release of the median nerve in the carpal tunnel, using a direct approach”) and the ICD-10 diagnosis code G56.0 (“carpal tunnel syndrome”) over seven successive 30-day intervals.

All statistical analyses were performed using R statistical software (version 3.1.2) [19], with the “survival” package and the “clogit” function [20].

## Results

### Description of the study population

We analyzed 5,517,680 hospitalizations for a singleton live birth between January 1^st^, 2007, and December 31^st^, 2013. The mean ± SD maternal age is this study population was 29.5 ± 5.4 years. The mean gestational age at delivery was 39.1 ± 1.8 weeks, and the mean birth weight was 3.3 ± 0.5 kg. The major risk factors for VTEs during the postpartum period are summarized in Table 1.

**Table 1:** Risk factors for VTEs during inpatient stays with delivery from 2007 to 2013

Variable	*n* = 5,517,680^a^
Pre-term delivery	12.8%^b^ (*n* = 475,935)
Maternal age >35 years	14.4% (*n* = 798,074)
Preeclampsia or eclampsia	4.1% (*n* = 229,853)
Morbid obesity (BMI ≥40 kg/m^2^)	0.3% (*n* = 18,871)
Cesarean section	19.6% (*n* = 1,084,005)
Postpartum hemorrhage	3.6% (*n* = 201,338)
Postpartum infection	1.4% (*n* = 77,968)
Birth weight <2.5 kg	5.8%^c^ (*n* = 266,174)

^a^ The results have been truncated to one decimal place

^b^ 1,333,025 missing values (collection was mandatory from 2009 onwards only)

^c^ 952,821 missing values (collection was mandatory from 2009 onwards only)

**Fig. 2:**
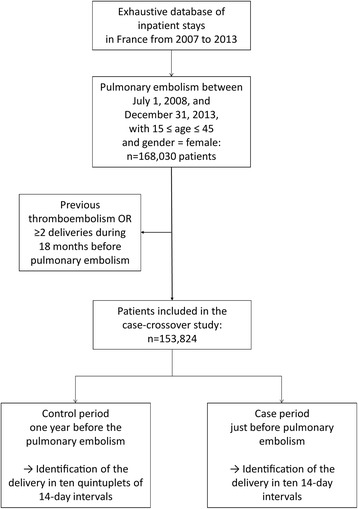
Flow diagram for patient inclusion

The flow diagram in Fig. 2 provides information on the inclusion of cases with pulmonary embolism. For the main case-crossover analysis, 153,824 patients with pulmonary embolism were included from July 1^st^, 2008, to December 31^st^, 2013. The mean number of VTEs per delivery was calculated with the value of 3,566,375 inpatient stays with delivery as a denominator.

### Risk estimation for each 2-week interval

Table 2 shows the number of deliveries during the case period and the mean number of deliveries during the five control periods for each time period. The rate of events per 100,000 deliveries and the ORs [95%CI] obtained for each 2-week interval are also shown.

**Table 2:** The risk of a VTE during the postpartum period, as a function of the time after delivery

Time period (days after delivery)	Case period Number of events ^b^ (rate/100,000 deliveries)	Control periods Number of events ^a, b^ (rate/100,000 deliveries)	OR ^b^ [95%CI]
0–13	387 (10.8)	22.4 (0.6)	17.2 [14.0–21.3]
14–27	259 (7.2)	23.0 (0.6)	11.2 [9.0–14.0]
28–41	139 (3.8)	22.6 (0.6)	6.1 [4.7–7.8]
42–55	85 (2.3)	22.0 (0.6)	3.8 [2.9–5.1]
56–69	62 (1.7)	22.8 (0.6)	2.7 [1.9–3.7]
70–83	48 (1.3)	24.4 (0.6)	1.9 [1.4–2.7]
84–97	36 (1.0)	25.4 (0.7)	1.4 [0.9–2.0]
98–111	31 (0.8)	27.6 (0.7)	1.1 [0.7–1.6]
112–125	24 (0.6)	25.6 (0.7)	0.9 [0.6–1.4]
126–139	24 (0.6)	27.2 (0.7)	0.8 [0.5–1.3]

^a^ The mean of the five control periods

^b^ The results have been truncated to one decimal place

**Fig. 3:**
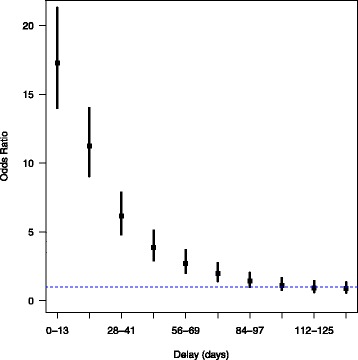
The risk of a VTE as a function of the time (in days) after delivery. The horizontal blue line corresponds to an OR of 1, and the error bars indicate the OR’s 95%CI

For postpartum weeks 1 to 2, 387 cases were identified, with a rate of 10.8/100,000 deliveries and an OR [95%CI] of 17.2 [14.0–21.3]. For postpartum weeks 3–4, the number of cases was 259, the rate was 7.2/100,000 and the OR [95%CI] was 11.2 [9.0–14.0]. We observed a progressive decrease in the risk of postpartum VTE over time, since the OR [95%CI] fell from 6.1 [4.7–7.8] for weeks 5 to 6 to 1.9 [1.4–2.7] for weeks 11 to 12. After 5 to 6 weeks, the risk of postpartum VTE was not increased beyond this time point. Figure 3 shows the risk curve; it can be seen that the risk of a VTE decays exponentially after delivery.

The post-hoc conservative analysis yielded slightly lower ORs; for weeks 7 to 8, weeks 9 to 10, and weeks 11 to 12, the ORs [95%CI] were respectively 3.0 [2.1–4.2], 1.9 [1.3–2.7] and 1.4 [0.9–2.0]. The results for the negative control are presented in the Additional file 1, the corresponding odds-ratios were systematically close to 1.

## Discussion

Our present results showed that an elevated risk of a VTE was present for nearly 12 weeks after delivery. The shape of the risk curve suggests that this risk decreases exponentially over time. Beyond 12 weeks, the risk was no longer elevated.

The incidence of pulmonary embolism in our study is similar to that reported by Jacobsen et al. [9] (22.0 per 100,000 deliveries) and by Lindqvist et al. [21] (21 per 100,000 [estimated from reported data]), but is greater than the incidence reported by Gherman et al. [22]: 8 per 100,000 (estimated from reported data).

It is also of value to compare the ORs for the occurrence of a VTE for weeks 1 to 6, weeks 7 to 12 and beyond 12 weeks. In Table 3, we report the ORs computed on our data, for the corresponding 6-week intervals (three control groups were used in this case). For the first 6 weeks, our results are close to those published by Kamel et al., but lower than those found by Heit et al. [4] (estimated by Jackson et al. [7] from reported data) or Pomp et al. [5]. For weeks 7 to 12 after delivery, our results are close to those published by Kamel et al., but lower than those found by Heit et al. [4], and higher than those found by Pomp et al. [5]. Finally, we found that the risk was no longer elevated 12 weeks after delivery: This agrees with Kamel et al. [6], who reported an OR [95%CI] of 1.4 [0.8–2.3] for weeks 13 to 18 and 0.8 [0.5–1.2] for weeks 19 to 24.

**Table 3:** Odds ratios reported by other studies, by 6-week intervals

	weeks 1–6	weeks 7–12	13–18 weeks
	Odds ratio [95%CI]	Odds ratio [95%CI]	Odds ratio [95%CI]
Heit et al. [4] (Jackson et al. [7])	21.5	0.69	-
Pomp et al. [5]	84.0 [31.7–222.6]	8.9 [1.7–48.1]	-
Kamel et al. [6]	12.1 [7.9–18.6]	2.3 [1.4–3.6]	1.4 [0.8–2.3]
Present study	11.3 [9.7–13.2]	2.6 [2.1–3.1]	1.1 [0.9–1.5]

Our use of a crossover design can be justified in two respects. Firstly, this design gave us more statistical power. Secondly, rigorous empirical evaluation by the OMOP has demonstrated that crossover design are superior in pharmaco-epidemiological studies [9] - particularly when compared with “new user”-type cohorts [23] and case-control studies [24]. The OMOP also showed that crossover cohort and case-crossover designs had similar methodological quality; these two cross-over designs were applied to data initially collected in a retrospective cohort. The use of these designs requires short exposure periods and events that have short durations and brief effects; these conditions were met in the present study. Finally, the results obtained in the complementary analysis, showing a greater risk of thrombosis after caesarean delivery, are in line with the results of Morris et al. [25], and in line with the most recent recommendations for thromboprophylaxis in United States (National Partnership for Maternal Safety) [26] and United Kingdom (Royal College of Obstetrician and Gynaecologists) [27]; However, the results of this additional analysis are not adjusted (in this case, the analysis compares groups of patients with each other -with or without cesarean section-, and no more the patient to herself) and no evaluation of the benefit-risk balance of thromboprophylaxis was conducted: For these reasons, these additional results do not assess the value of such thromboprophylaxis after cesarean.

The present study had several limitations. Firstly, it is also well known that survival bias can influence the findings from observational, pharmaco-epidemiological studies [28–30], since the inclusion criteria can sometimes lead to the selection of low-risk patients. We cannot rule out the presence of survival bias because all patients with a VTE between January 1^st^, 2007 and June 30^th^, 2008 were excluded. Secondly, the use of a statistical test for each 2-week interval inevitably increases the type I error. This may have biased our estimates of the time point beyond which the risk of postpartum pulmonary embolism is no longer elevated, but not the temporal decrease in pulmonary embolism incidence. Thirdly, the use of hospital administrative databases always raises the question of data accuracy [31, 32]. However, the codes we used for pulmonary embolism and delivery are both known to be associated with a good level of recall. In addition, it seems reasonable to consider that misclassification of VTE may occur in both case and control periods, which would not necessarily change the odds ratios and could thus be a non-differential bias. Fourthly, some dates of delivery could be inaccurate, since this date has only been mandatory since 2010. Before 2010, the database’s default delivery date is set to zero and so it is (sometimes wrongly) considered that delivery occurs on the first day of hospitalization. To some extent, this choice may have artificially lengthened the time period between delivery and the occurrence of a VTE. Furthermore, the analysis of events that only account for a proportion of the total events of interest constitutes another study limitation, and raises the question of whether our findings can be generalized to the entire set of events concerned. In the case of VTEs, it is unclear whether PE events are a good proxy for deep venous thromboses [33]. In addition, it is possible that some massive pulmonary embolisms, leading to death without prior hospitalization, have not been detected. Finally, although our database is comprehensive for hospitalizations in France, some patients who gave birth in France could then have been lost to follow-up (e.g. emigration).

Lastly, we did not perform additional subgroup analyses as a function of the presence of anticoagulation therapy, since this information was not available in the database. It would be of value to evaluate the risk of bleeding associated with anticoagulation therapy. This could be performed by analyzing a drug prescription database. Measures of association, computed in our study, do not account for the use of mechanical or pharmacological VTE prophylaxis: It is likely that the measures of association computed for the first 6 weeks are modified by the use of prophylaxis in our population; Contrariwise, it seems reasonable to think that our estimation beyond 6 weeks is less modified. In addition, the presence of a prophylaxis beyond 6 weeks (for some patients in our analyzed sample) would be conservative regarding the results obtained for the intervals beyond 6 weeks, as it would decrease the risk of a pulmonary embolism.

## Conclusions

An elevated risk of a postpartum pulmonary embolism is present for nearly 12 weeks after delivery, and appears to decay exponentially. In groups at high risk of a VTE, it may be of value to assess the relevance of extending the duration of preventive anticoagulation therapy beyond 6 weeks. To adequately assess the risks vs benefits of extended anticoagulation therapy after delivery, comparative effectiveness studies in large populations are needed.

## Additional file

Additional file 1:
**Appendix 1.** Venous thromboembolic risk after release of the median nerve in the carpal tunnel (negative control). **Appendix 2.** ICD-10 codes to identify pulmonary embolism. **Appendix 3.** Definition of pulmonary embolism including a procedure code. **Appendix 4.** Exclusion criteria. **Appendix 5.** Additional analysis to compare the risk of pulmonary embolism after cesarean section with the risk of pulmonary embolism after vaginal delivery. (DOCX 124 kb)
