# Supplementary material for: Case-crossover study to examine the change in postpartum risk of pulmonary embolism over time
Source: BMC Pregnancy Childbirth. 2017 Apr 14;17:119. doi: 10.1186/s12884-017-1283-y (PMC5391590; doi:10.1186/s12884-017-1283-y)
Supplement: Additional file 1: — Appendix 1. Venous thromboembolic risk after release of the median nerve in the carpal tunnel (negative control). Appendix 2. ICD-10 codes to identify pulmonary embolism. Appendix 3. Definition of pulmonary embolism including a procedure code. Appendix 4. Exclusion criteria. Appendix 5. Additional analysis to compare the risk of pulmonary embolism after cesarean section with the risk of pulmonary embolism after vaginal delivery. (DOCX 124 kb) [file 12884_2017_1283_MOESM1_ESM.docx]

Additional file

# Appendix 1: Venous thromboembolic risk after release of the median nerve in the carpal tunnel (negative control)

Table 1 – The risk of a venous thromboembolic event after release of the median nerve in the carpal tunnel, as a function of the time

| **Time period (days after surgery)** | **Case period**  **Number of events*** | **Control period**  **Number of events*** | **OR***  **[95%CI]** |
| --- | --- | --- | --- |
| **0-29** | 44 | 32 | 1.3 [0.8-2.1] |
| **30-59** | 33 | 33 | 1.0 [0.6-1.6] |
| **60-89** | 30 | 27 | 1.1 [0.6-1.8] |
| **90-119** | 40 | 34 | 1.1 [0.7-1.8] |
| **120-149** | 23 | 32 | 0.7 [0.4-1.2] |
| **150-179** | 37 | 33 | 1.1 [0.7-1.7] |
| **180-209** | 33 | 31 | 1.0 [0.6-1.7] |

*results have been truncated to one decimal place

# Appendix 2: ICD-10 codes to identify pulmonary embolism

The ICD-10 codes used to identify pulmonary embolism are I26.0 (“Pulmonary embolism with acute cor pulmonale”), I26.9 (“Pulmonary embolism without acute cor pulmonale”) and the specific obstetrical code O88.2 (“Obstetric thromboembolism”). The latter code encompasses many other anomalies, and thus is not specific for pulmonary embolism. Furthermore, our preliminary analysis of the French national inpatient database showed that the ICD-10 code O88.2 was always combined with I26.0 or I26.9 whenever a CCAM code for the diagnosis of pulmonary embolism (ZBQH001, ECQH010, ECQH011, DZQM005 and GFQL0xx) was present (data not shown). Accordingly, we decided to use both I26.0 and I26.9 (but not O88.2) to identify VTEs.

# Appendix 3: Definition of pulmonary embolism including a procedure code

Procedure CCAM codes used to define the pulmonary embolism (in addition to I26.* code): EHNE001, EHNE002, HEQE002, ZCQH001, HESE002, HHQE005, HHQE002, HJQE001, HHFE002, HHFE004.

# Appendix 4: exclusion criteria

Patients who had been admitted to hospital for a VTE between January 1st, 2007, and June 30th, 2008 (i.e. before the beginning of the inclusion of the patients with pulmonary embolism from July 1st, 2008), were excluded because we wanted to be sure that (i) no other VTE occurred during the study (and at least 3 months before).

The codes used to detect previous VTE were I26.* (“Pulmonary embolism”) and I80.* (“Phlebitis and thrombophlebitis”). Lastly, patients who had given birth several times during the 18 months preceding the VTE were also excluded (code Z37.*, “Outcome of delivery”).

# Appendix 5: Additional analysis to compare the risk of pulmonary embolism after cesarean section with the risk of pulmonary embolism after vaginal delivery

For this purpose, we conducted two analyses with a modified definition of the exposure: (i) single live birth AND cesarean section and (ii) single live birth AND non cesarean section. We used exactly the same criteria as in the main analysis, only the definition of the exposure changed:

- Postpartum risk of pulmonary embolism after cesarean section: to define the exposure of interest, we added the following criterium “AND CCAM code LIKE ‘JQGA%’ ” (Figure 1)
- Postpartum risk of pulmonary embolism after vaginal delivery: to define the exposure of interest, we added the following criterium “AND NO CCAM code LIKE ‘JQGA%’ ” (Figure 2)


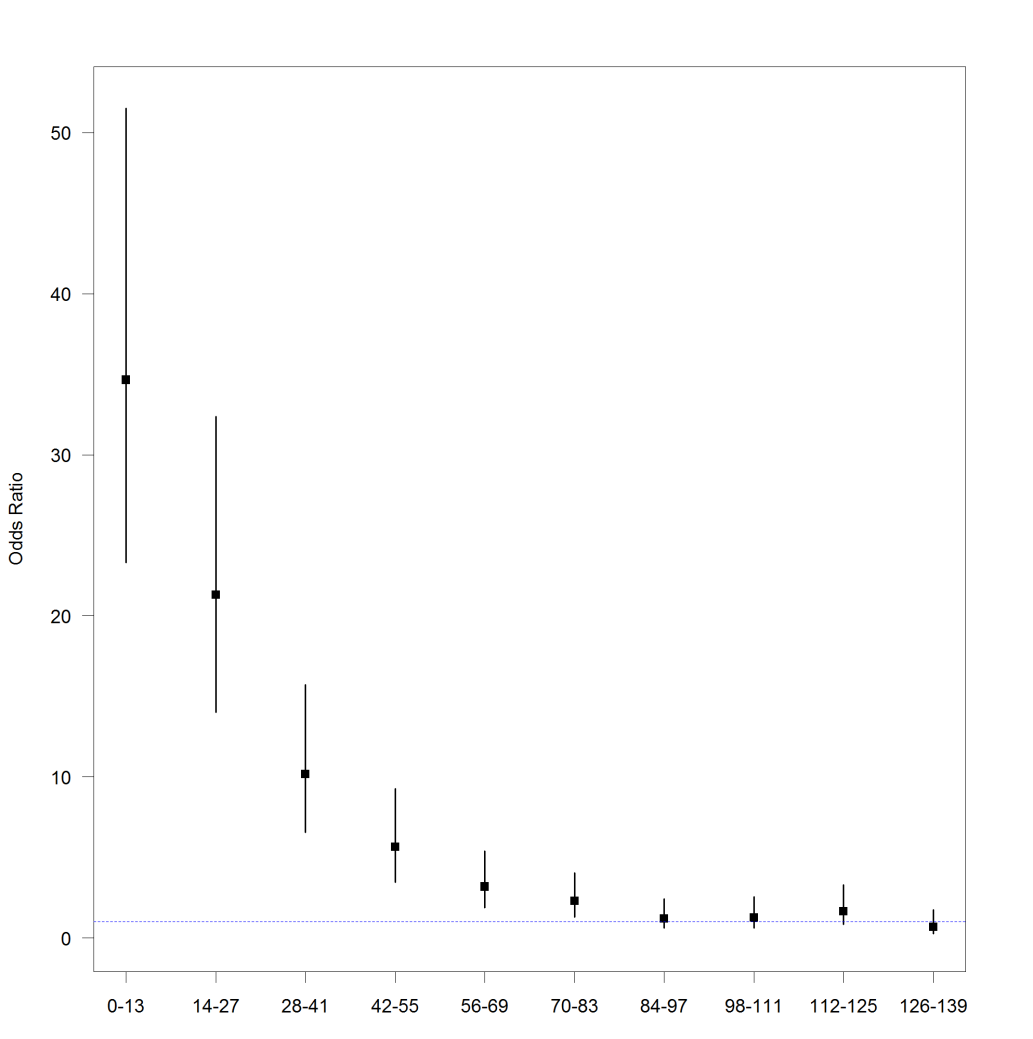


Figure 1 - The postpartum risk of pulmonary embolism over time after cesarean section


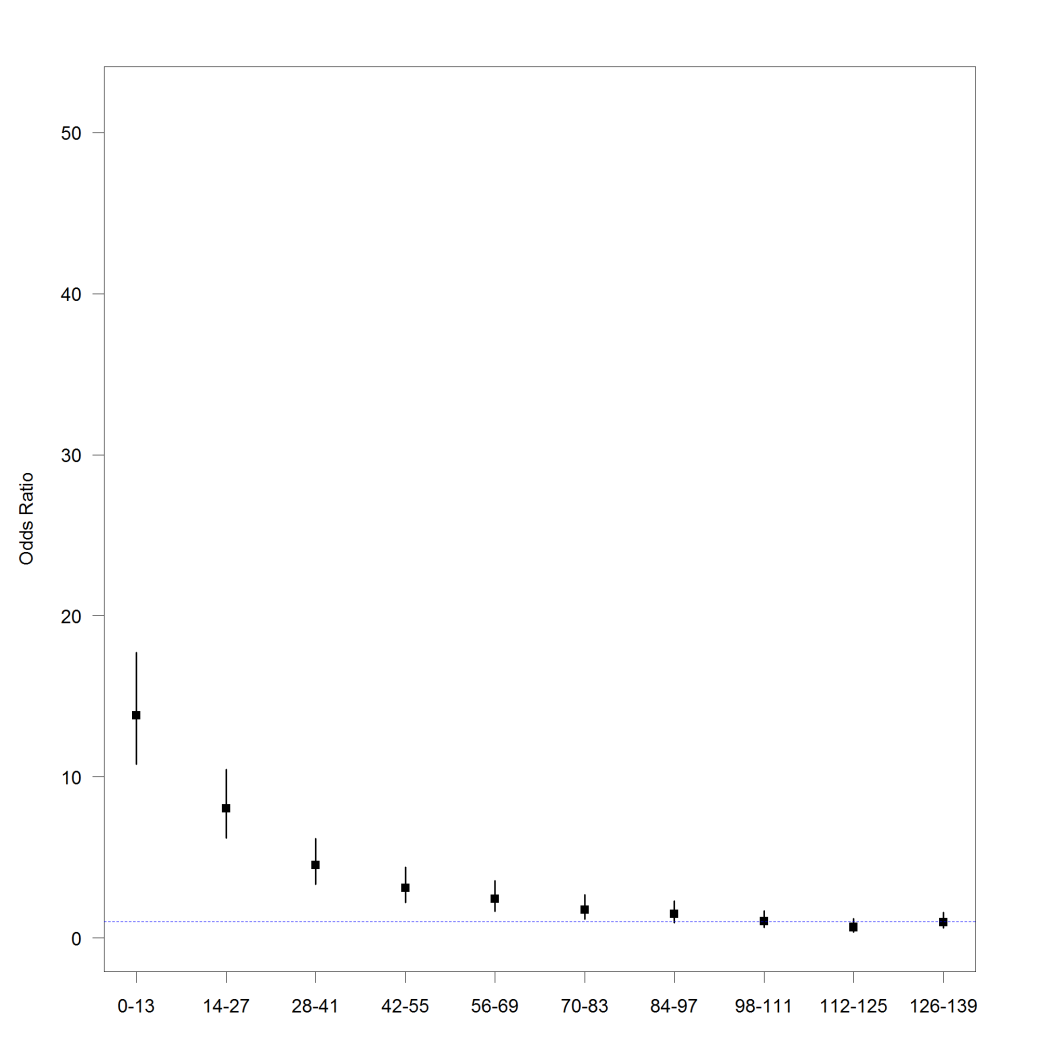


Figure 2 - The postpartum risk of pulmonary embolism over time after vaginal delivery
